# Percutaneous Chevron Osteotomy: A Prospective Randomized Controlled Trial

**DOI:** 10.3390/medicina58030359

**Published:** 2022-03-01

**Authors:** Serban Dragosloveanu, Viola Maria Popov, Dragoș-Corneliu Cotor, Christiana Dragosloveanu, Cristian Ioan Stoica

**Affiliations:** 1 Faculty of medicine, “Carol Davila” University of Medicine and Pharmacy, 020021 Bucharest, Romania; serbandrago@gmail.com (S.D.); violamariap@gmail.com (V.M.P.); chrisitanacelea@gmail.com (C.D.); cristi.stoica@foisor.ro (C.I.S.); 2 Department of Orthopaedics, “Foisor” Orthopaedics Hospital, 020021 Bucharest, Romania; 3 Colentina Clinical Hospital Hematology Clinic, 020021 Bucharest, Romania

**Keywords:** hallux valgus, osteotomy, percutaneous

## Abstract

*Introduction*: Minimally invasive surgical techniques for hallux valgus have gained popularity, showing good results characterized by smaller postoperative scars, less pain, lower infection risk, and fewer wound complications. Given the lack of evidence available in our country regarding this subject, especially about this type of surgical technique, our paper aims to compare open and MIS approaches for chevron osteotomy. We evaluated the outcome and complications after 12 months. *Materials and Methods*: We undertook a prospective, randomized, controlled, single-center study between October 2017 and December 2020. The patients were randomized into two groups: one group that received percutaneous chevron osteotomy (MIS), and the other, open chevron osteotomy (OC). For clinical assessment, we determined the function and the level of pain using the Visual Analogue Scale (VAS) and The American Orthopaedic Foot and Ankle Surgery score (AOFAS). The VAS scale was measured before the surgical procedure, at discharge, and at 3 weeks, 6 weeks, 6 months, and 12 months after surgery. The AOFAS score was calculated preoperatively and after 6 months. The hallux angle (HVA) and intramedullary angle (IMA) were measured preoperatively, and at 6 weeks, 6 months and 12 months. *Results*: We included 26 cases in the open chevron osteotomy group (24 female, 2 male) and 24 in the MIS group (24 female, 0 male). Both groups demonstrated improvements regarding the IMA and HVA at the last follow-up without any significant differences between the groups at the final assessment. The VAS showed significantly better post-operative results for the MIS group at discharge (*p* < 0.001) and 3 weeks (*p* < 0.001), 6 weeks (*p* < 0.001), and 6 months (*p* = 0.004) post-surgery. The AOFAS showed no significant differences either before or after surgery. Four cases with screw prominence were reported, three of which belonged to the MIS group. Only one case with metatarsalgia was found in the OC group. *Conclusions*: This paper demonstrates that minimally invasive chevron osteotomy has comparable results with open chevron osteotomy, even though surgical time and radiological exposure are significantly longer. More studies are required to evaluate the complications and the risk of recurrences.

## 1. Introduction

Hallux valgus is a very common foot deformity usually caused by multiple factors that sometimes require surgical treatment. According to the literature, patients with foot pathologies have a worse quality of life related to foot health [1,2]. It also seems that older people with poorer foot health are linked with increasing severity of hallux valgus [1]. Females are usually more predisposed to this kind of deformity, especially in cases that have a family history. Another risk is constricting footwear usage [3]. A recent article described the morphology of the first metatarsal as an additional risk factor for the occurrence of hallux valgus [4]. A longer first metatarsal has a higher risk of developing a deformity. Additionally, a longer lateral aspect of the first metatarsal than the medial aspect of the bone was linked to a higher incidence of hallux valgus [4]. At least one hundred open surgical techniques have been described in many different papers [5,6,7]. The most popular techniques described are Scarf, Ludloff, and chevron. All three produce favorable outcomes [8,9,10,11,12]. Despite these good results, many cases develop complications such as pain or stiffness [13,14,15,16].

Minimally invasive surgery (MIS) and percutaneous surgical techniques have gained popularity, showing good results characterized by smaller postoperative scars, less pain, lower infection risk, and fewer wound complications. The soft tissues surrounding the structures may influence the mobility of the metatarsophalangeal joint [17,18,19]. These techniques have gained popularity in the last few decades, especially during the 1990s and 2000s [20,21,22,23]. One of the first minimally invasive techniques was developed by Isham [24]. He developed a percutaneous adductor hallucis release and a percutaneous closing wedge osteotomy without any internal fixation. Even though this kind of procedure could fail due to instability, he reported good results [24,25]. A second-generation osteotomy was described by Bosch based on Hohmann osteotomy [6,26]. It consisted of a short osteotomy at the metatarsal neck fixed with a K-wire inserted in the proximal canal [7]. Despite the K-wire fixation, instability at the osteotomy site has been reported [27,28,29]. In order to overcome this complication, a minimally invasive chevron osteotomy was developed, which could be fixed using compression screws. This technique demonstrated better and more predictable results [30]. Despite the favorable outcomes reported in the literature [23,31,32], there are still controversies regarding this topic. Some articles have reported a lack of evidence regarding the efficiency of the percutaneous osteotomy [33,34,35]. Mild and moderate cases are most likely the best candidates for an MIS procedure.

Our paper aims to compare open and MIS approaches for chevron osteotomy. We wanted to evaluate the outcome and complications after 12 months.

## 2. Materials and Methods

Even though there are papers available that compare percutaneous techniques with open osteotomies, we did not find any evidence from our country on this subject. Thus, we decided that was more relevant to develop a prospective, randomized, controlled trial. The research was conducted between October 2017 and December 2020. The study was approved by the Ethics Committee of ‘Foişor’ Orthopaedics-Traumatology and Osteoarticular TB Hospital in Bucharest, Romania (no. 1153/2017, date of approval 5 February 2017). Informed consent was obtained from all participants included in the study. All procedures were conducted in accordance with national ethical standards and with the 1964 Helsinki declaration.

**Figure 1:**
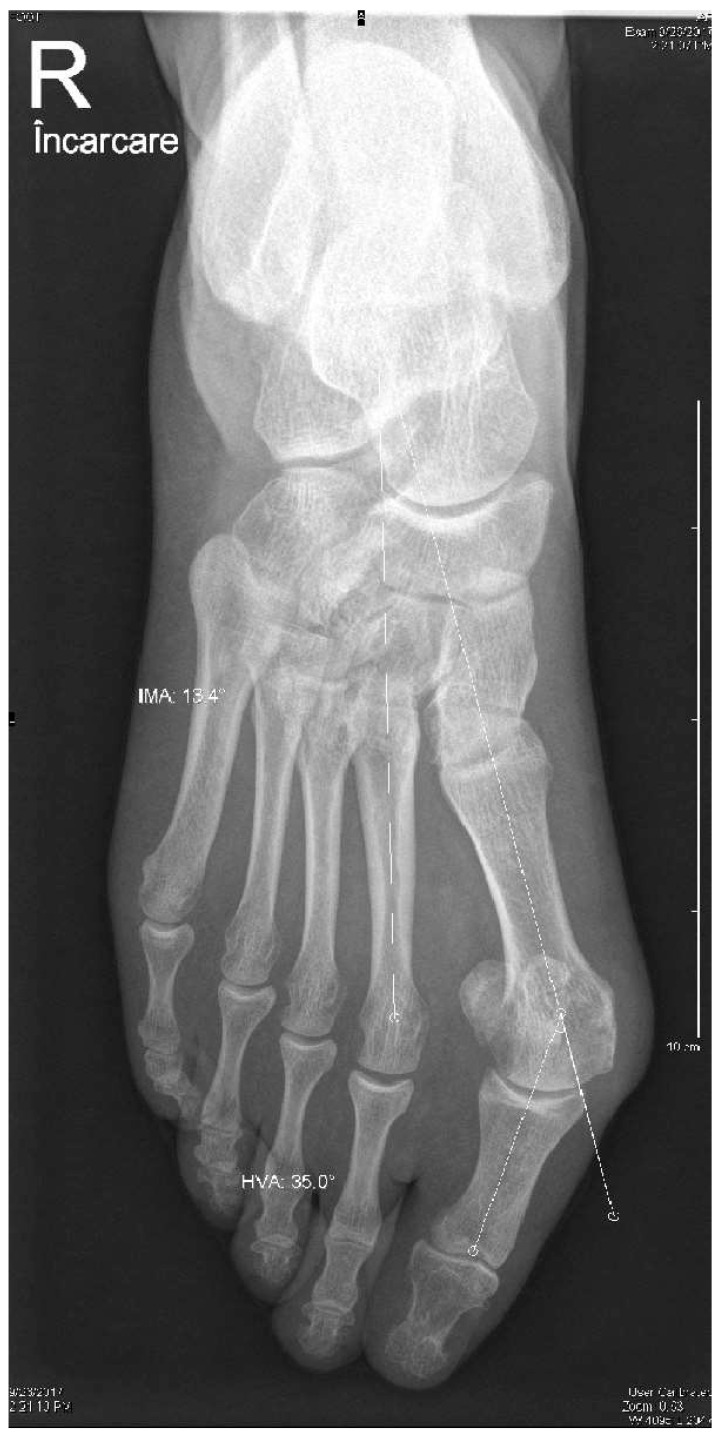
Preoperative weight-bearing anteroposterior image and measurements of HVA and IMA.

The patients were recruited according to the following inclusion criteria: patients older than 20 years old, failed conservative treatment, and moderate valgus deformity. The hallux valgus severity was preoperatively measured using weight-bearing X-rays, and moderate deformity was defined as a hallux valgus angle (HVA) between 20° and 40° and an intermetatarsal angle (IMA) between 11° and 16° (Figure 1) [36]. The HVA was measured between the axis of the first metatarsal and the longitudinal axis of the proximal phalanx of the first ray [37]. The IMA is formed by the longitudinal axes of the first and second metatarsals. The exclusion criteria applied were patients with previous first metatarsal osteotomy, instability of the first metatarsocuneiform, osteoarthritis of the metatarsophalangeal joint, and systemic diseases that may affect the musculoskeletal system (gout, rheumatoid arthritis, systemic lupus erythematosus, etc.).

The patients were randomized into two groups: one group that received a percutaneous chevron osteotomy (MIS) and another group that received an open chevron osteotomy (OC). We achieved the randomization using sealed envelopes prepared by an independent person. The envelopes were marked with the name of the type of the surgical procedure and then stored in a box. Before the surgery, an envelope was drawn. The envelope contained instructions regarding the surgical technique. For the clinical assessment, we determined the function and the level of pain using the Visual Analogue Scale (VAS) and The American Orthopaedic Foot and Ankle Surgery score (AOFAS) [38]. The VAS scale was measured before the surgical procedure, at discharge, and 3 weeks, 6 weeks, 6 months, and 12 months after the surgery. The AOFAS score was calculated preoperatively and after 6 months.

**Figure 2:**
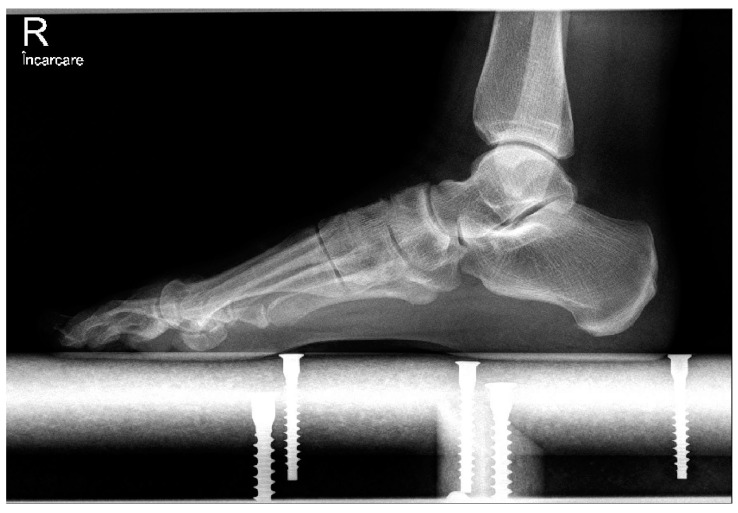
Preoperative lateral weight-bearing image.

We also radiologically evaluated each patient using standard weight-bearing anteroposterior and lateral images of the foot (Figure 1 and Figure 2). The HVA and IMA angles were preoperatively measured using Cedara I-View 6.3.3. At 6 weeks, 6 months, and 12 months, we performed additional radiological images to evaluate the angle modifications.

### 2.1. Operative Technique

For the percutaneous chevron osteotomy, the patient was placed in a supine position, and a tourniquet was placed on the thigh. First, a 4 mm dorsal incision was performed between the 1st and 2nd metatarsal heads, and the adductor tendon was released percutaneously. After the release, a 15 mm incision was performed on the dorsomedial side of the foot. Over 3 mm of the bunion was excised using a motor-driven reamer. A 60 degrees V-shaped osteotomy was performed under fluoroscopic guidance. The apex of the osteotomy was positioned 2 mm proximal to the anatomical center of the metatarsal head. After that, the head was displaced laterally, and a K-wire was inserted for provisional fixation. After radiological assessment, the osteotomy was fixed percutaneously with one 3.0 mm cannulated screw placed from proximal to distal, and the K-wire was removed. The wound was then sutured, and a bandage was applied.

**Figure 3:**
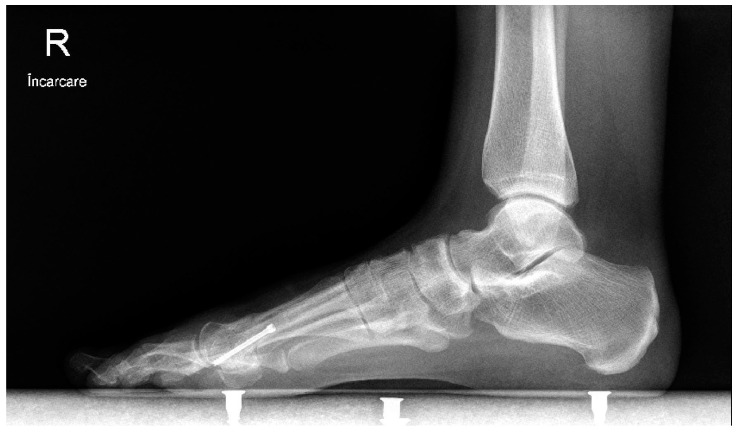
Lateral weight-bearing image at 12 months after surgery.

**Figure 4:**
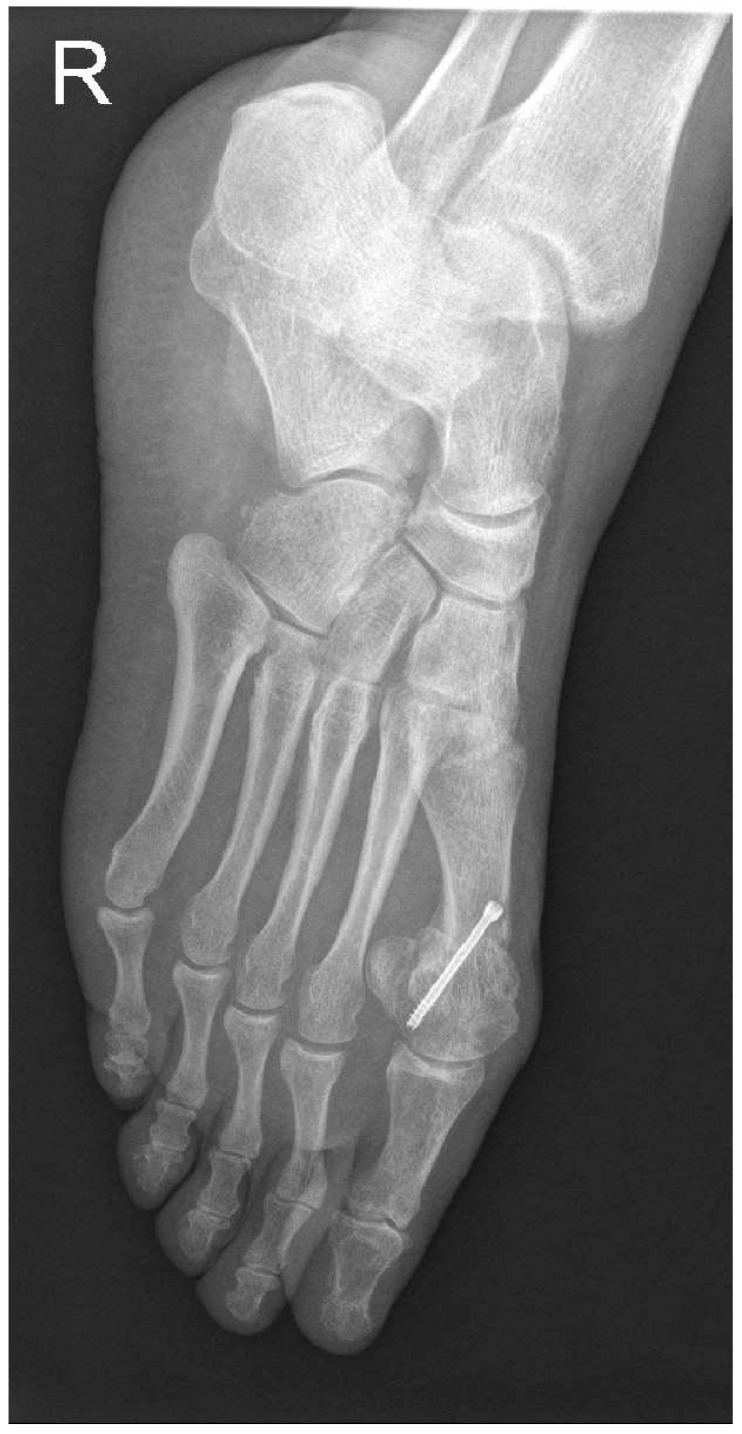
Postoperative oblique image after 12 months.

**Figure 5:**
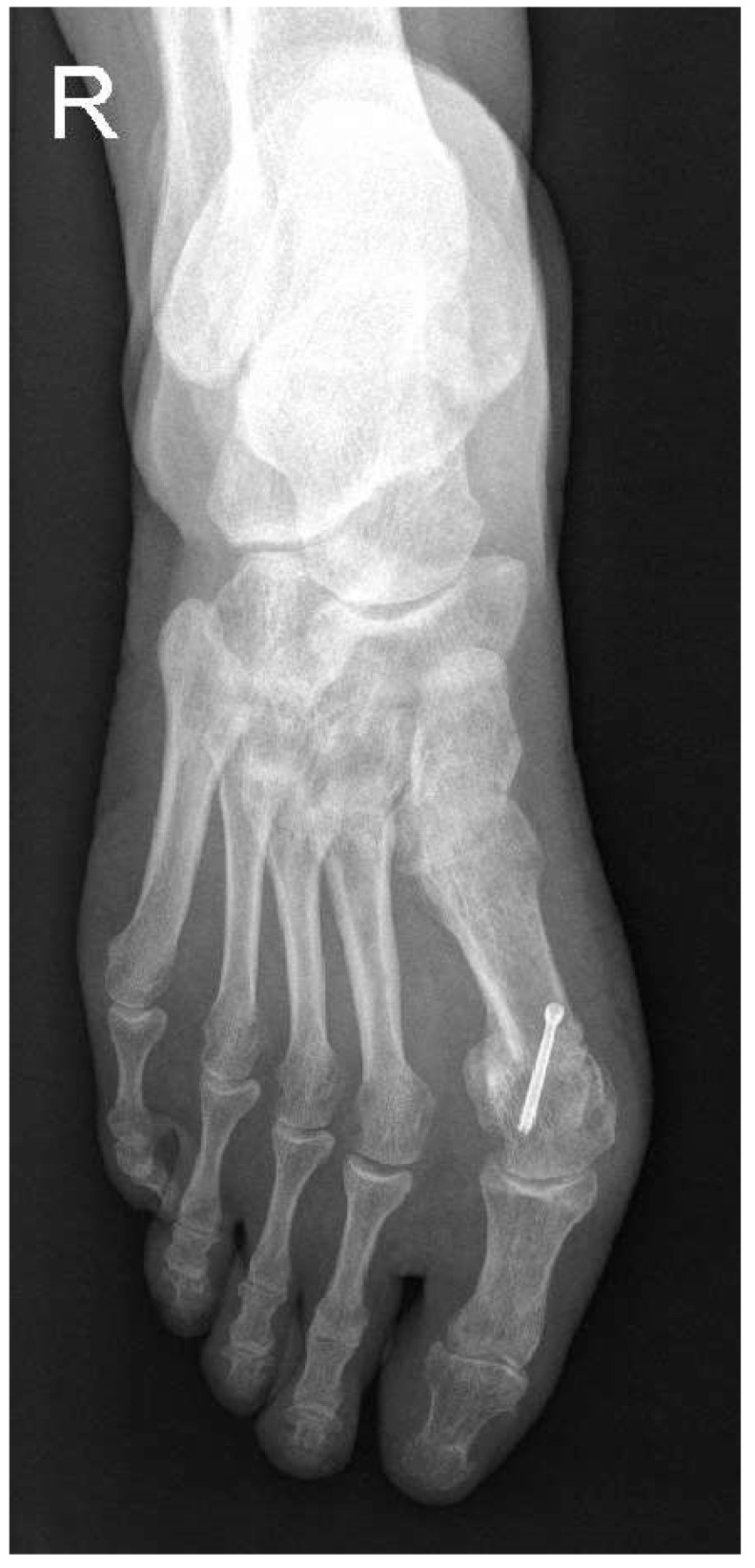
Postoperative weight-bearing anteroposterior image after 12 months.

In the open chevron osteotomy group, a 5 cm dorsomedial incision was made. The V-shaped osteotomy was performed using a motor-driven saw. The osteotomy was created in 60 degrees with an angled chevron, and the tip of the osteotomy was positioned 2 mm proximal to the anatomical center of the metatarsal head. The metatarsal head was repositioned in a lateral direction and directly inspected through the incision. The osteotomy was then fixed using a 3.0 mm cannulated screw. The adductor hallucis tendon was released from the fibular sesamoid through a 15 mm dorsal incision. For both groups, the skin was closed using separate 2-0 sutures, and a soft bandage was applied. Postoperative treatment consisted of soft dressings applied weekly. After 3 weeks, the sutures were removed. Full weight-bearing on the forefoot was avoided by wearing an orthosis for six weeks (Figure 3, Figure 4 and Figure 5).

### 2.2. Data Analysis

We presented as means and standard deviations (±SD) the continuous variables. In order to evaluate the differences between preoperative and postoperative clinical measurements, a t test was performed. For the continuous variables, Student’s t test or a Mann–Whitney U test was performed. For the assessment of differences in proportions of categorical values, we used the Pearson chi-square test. A *p* value lower than 0.05 was noted as significant. The statistical analyses were performed by an independent statistician using SPSS version 27.0.

### 2.3. Sample Size

We established a minimal clinically relevant difference of at least 15 in the AOFAS score. Another input was a standard deviation of 15 points, an alpha risk of 5%, and a beta of 0.1. For these parameters, at least 46 patients had to be included.

## 3. Results

We included 26 cases in the open chevron osteotomy group (24 female, 2 male) and 24 in the MIS group (24 female, 0 male). The distribution of sexes did not demonstrate any statistically significant differences(*p* = 0.490). The average age was 49 ± 15 for the MIS group and 55 ± 15 for the OC group (*p* = 0.155) (Table 1).

From a radiological standpoint, both groups demonstrated improvements regarding the IMA and HVA at the last follow-up. We did not find any significant differences between the groups at the final assessment (Table 2).

Regarding the clinical outcome, we found significant improvements in the VAS and AOFAS score for both groups (*p* < 0.001). The VAS showed significantly better postoperative results for the MIS group at discharge (*p* < 0.001), 3 weeks (*p* < 0.001), 6 weeks (*p* < 0.001), and 6 months (*p* = 0.004). At the last follow-up, we found the pain level was comparable between both groups. (*p* = 0.285). Regarding AOFAS results, we did not notice any significant differences between the groups, either before surgery (0.134) or at the last follow-up (0.125). The osteotomy consolidated in 13.12 ± 3.3 weeks for the MIS group and 13.25 ± 2.9 weeks for the open chevron osteotomy group and showed no statistically significant differences (*p* = 0.882).

We had five complications reported in our study. Soft tissue irritation caused by screw prominence occurred in three cases from the MIS group and one case from the OC group. The screw caused local pain, and a second procedure for hardware removal was necessary after 3 months. One case from the open chevron osteotomy group reported metatarsalgia after 6 months. No wound or septic complications were recorded. The average radiological screen time was significantly longer for the MIS group (15.5 ± 5.6 s) compared to the second cohort (1.8 ± 3.8 s) (*p* < 0.001).

## 4. Discussion

This prospective randomized controlled study compares minimally invasive chevron osteotomy with the open chevron technique. The most important finding is that the results were comparable between the groups, with significant improvements regarding the clinical outcome. The main objective of hallux surgery is to achieve the realignment of the metatarsophalangeal joint and to achieve a painless forefoot [39]. In terms of IMA and HVA, we found significant improvements and no loss in correction. The IMA is an important parameter to evaluate the amount of correction possible during the osteotomy. This angle also has a strong correlation with the sesamoid position, even after the surgical procedure [40,41,42]. A satisfactory IMA correction demonstrates whether the metatarsal head is sufficiently shifted laterally after the osteotomy. In our study, the angle correction after the MIS procedure was comparable with that after the open chevron osteotomy. Our results are supported by other reports found in the literature [24,42,43,44]. Metatarsal osteotomy also has an indirect impact on HVA correction. The main impact on this angle is lateral abductor release. The HVA showed a significant correction but with comparable results between both groups. Our results are also supported by other available results [23,42]. The abovementioned results demonstrate that the percutaneous approach is capable of restoring the alignment of the greater toe.

Hallux valgus severity is linked to foot health and quality of life, especially in elderly people [1,2]. Thus, the improvement of quality of life is one of the aims of the surgical procedure. AOFAS scores demonstrated significant functional improvements for both groups. Neither groups showed any statistically significant differences after 6 months. This finding is supported by other retrospective studies available in the literature [42,43,44]. The VAS score showed significant improvements for both groups. The MIS technique showed significantly decreased pain levels at discharge, at 3 weeks, 6 weeks, and at 6 months. Despite this result, after 12 months, the VAS scores showed no significant differences. For this reason, we do not think that it is clinically significant in the long term. We also think that pain levels may not influence the functional results. According to Chen et al., residual pain can be identified 6 months after surgery. After that, the pain level starts decreasing for the following year and a half [42,45]. The difference in early postoperative VAS results could be linked to the minor soft tissue dissection when the MIS technique is applied. According to Kaufmann et al., this also demonstrated better patient satisfaction at 3-month follow-up [42].

We report soft tissue irritation in one case with open chevron osteotomy and three cases with MIS osteotomy. All cases required another surgical procedure in order to remove the screw. The reason for tissue irritation could be the oblique insertion and slight protrusion of the screw head. Only one case reported a transfer metatarsalgia after the open chevron osteotomy. It seems that shortening of the first metatarsal during the osteotomy could be the main reason for this complication [46].

The radiological screen time was significantly longer for the MIS group. This result could be due to the fact that the MIS technique is more demanding than the open Chevron osteotomy and may also influence the surgical time, depending on the surgeon’s experience with the surgical technique, —which has also been described by other reports [47,48].

The most important limitation of our study is the sample size. A bigger cohort and a longer follow-up is required to further evaluate the results with a higher statistical power. Additionally, a larger number of patients would help us evaluate whether the MIS approach produces more complications than open chevron osteotomy The small number of participants was caused by the limited resources available in our clinic. In addition, a longer follow-up would help us further evaluate the risk of recurrences. Another limitation in our study is the fact that all cases were performed by a single surgeon. It would be ideal to compare this treatment method performed by more surgeons for more reliable data. Since a variety of minimally invasive techniques have gained popularity, we find this study clinically relevant. The majority of papers we found in the literature were systematic reviews; thus, we consider that a larger prospective randomized study from our clinic would be beneficial.

## 5. Conclusions

This paper demonstrated that, after 12 months, a minimally invasive chevron osteotomy has comparable functional and radiological outcomes with open chevron osteotomy, even though the surgical time and the radiological exposure could be significantly longer. We consider that the hallux valgus treatment should depend on the patient characteristics and surgical experience. A more detailed study with a larger sample is necessary to evaluate the complications and the risk of recurrences.

## Figures and Tables

**Table 1:** Baseline characteristics.

	MIS Chevron	Open Chevron	*p* Value
Sex (f/m)	24/0	24/2	0.490
Age (years ± SD)	49.4 ± 15.3	55.3 ± 13.6	0.155
Side (left/right)	16/8	11/15	0.098

**Table 2:** Clinical data of both groups.

		MIS Group	OC Group	*p* Value
IMA	Preoperative	15.1 ± 1.8	15.6 ± 1.9	0.345
	6 weeks	8.1 ± 2.1	7.9 ± 1.8	0.718
	6 months	7.9 ± 1.4	7.6 ± 1.1	0.401
	12 months	7.2 ± 1.8	6.4 ± 1.5	0.093
HVA	Preoperative	32.5 ± 2.5	31.9 ± 4.3	0.553
	6 weeks	10.9 ± 3.1	10.2 ± 3.9	0.488
	6 months	9.6 ± 2.4	9.5 ± 1.8	0.867
	12 months	8.8 ± 3.1	8.9 ± 2.3	0.896
VAS	Preoperative	7.6 ± 1.2	7.1 ± 1.8	0.257
	Discharge	2.5 ± 0.8	4.5 ± 1.4	<0.001
	3 weeks	1.4 ± 0.5	2.8 ± 0.9	<0.001
	6 weeks	0.4 ± 1	2.0 ± 0.8	<0.001
	6 months	0.2 ± 0.8	0.8 ± 0.6	0.004
	12 months	0.2 ± 0.6	0.4 ± 0.7	0.285
AOFAS	Preoperative	65.7 ± 3.8	61.4 ± 4.5	0.134
	6 months	85.6 ± 4.1	79.4 ± 3.6	0.125

## Data Availability

Not applicable.
